# MIMO-Uformer: A Transformer-Based Image Deblurring Network for Vehicle Surveillance Scenarios

**DOI:** 10.3390/jimaging10110274

**Published:** 2024-10-31

**Authors:** Jian Zhang, Baoping Cheng, Tengying Zhang, Yongsheng Zhao, Tao Fu, Zijian Wu, Xiaoming Tao

**Affiliations:** 1 China Mobile (Hangzhou) Information Technology Co., Ltd., Hangzhou 311100, China; zhangjianhz@cmhi.chinamobile.com (J.Z.); cbp21@mails.tsinghua.edu.cn (B.C.); zhangtengying@cmhi.chinamobile.com (T.Z.); wuzijian@cmhi.chinamobile.com (Z.W.); 2 Department of Electronic Engineering, Tsinghua University, Beijing 100084, China; taoxm@tsinghua.edu.cn

**Keywords:** vehicle-surveillance scenarios, image deblurring, transformer-based network, MIMO-UNet, IoP factor, supervised morphological loss

## Abstract

Motion blur is a common problem in the field of surveillance scenarios, and it obstructs the acquisition of valuable information. Thanks to the success of deep learning, a sequence of CNN-based architecture has been designed for image deblurring and has made great progress. As another type of neural network, transformers have exhibited powerful deep representation learning and impressive performance based on high-level vision tasks. Transformer-based networks leverage self-attention to capture the long-range dependencies in the data, yet the computational complexity is quadratic to the spatial resolution, which makes transformers infeasible for the restoration of high-resolution images. In this article, we propose an efficient transformer-based deblurring network, named MIMO-Uformer, for vehicle-surveillance scenarios. The distinct feature of the MIMO-Uformer is that the basic-window-based multi-head self-attention (W-MSA) of the Swin transformer is employed to reduce the computational complexity and then incorporated into a multi-input and multi-output U-shaped network (MIMO-UNet). The performance can benefit from the operation of multi-scale images by MIMO-UNet. However, most deblurring networks are designed for global blur, while local blur is more common under vehicle-surveillance scenarios since the motion blur is primarily caused by local moving vehicles. Based on this observation, we further propose an Intersection over Patch (IoP) factor and a supervised morphological loss to improve the performance based on local blur. Extensive experiments on a public and a self-established dataset are carried out to verify the effectiveness. As a result, the deblurring behavior based on PSNR is improved at least 0.21 dB based on GOPRO and 0.74 dB based on the self-established datasets compared to the existing benchmarks.

## 1. Introduction

Security surveillance is essential for preserving human daily life. A vehicle is one of the most important targets for existing surveillance scenarios. However, when the exposure time is too long and there is a fast relative motion between the camera and the target in the duration, motion blur will occur in the captured images [1]. Motion blur influences the quality of the captured images and stops the observer from receiving valuable information, resulting in visual information loss. Therefore, it is essential to propose a reliable method for motion deblurring under vehicle-surveillance scenarios. The motion blur of an image can be formulated as *B* = *K* * *S* + *N* [2], where *B* and *S*, respectively, represent the blurry and sharp image, and *N* is the observation noise. *K* is a large sparse matrix, and each column of *K* represents a blur kernel for the corresponding pixel in *S*. *K* is handled on *S* by the matrix multiplication operator *. The process of deblurring is to restore *S* given *B*, assuming that *K* and *N* are unknown. In former works [3,4,5], the blur kernel was firstly estimated using the observed samples, and the sharp image was recovered using the inverse operation. However, this kind of method needs some hand-crafted priors of *S* and *K*. If the priors are preset improperly, the blur kernel will be estimated incorrectly.

Recently, the methods based on a convolution neural network (CNN) have been introduced to the deblurring research. One kind of CNN-based method [6,7,8,9] firstly resorts to a CNN as the blur kernel estimator, and then, the sharp image is restored through the kernel-based deconvolution. Subsequently, some methods are proposed to directly restore the sharp image from the blurry one. A deblurring method based on a multi-stage CNN is proposed in [10]. The structure of the network is composed of multiple stacked sub-networks, and each sub-network handles it at a specific scale. The sharp image is step-by-step restored through each of the sub-networks, which is called a coarse-to-fine process. Inspired by this work, various coarse-to-fine methods have been gradually proposed [11,12,13] and remarkably improved the deblurring performance. But this coarse-to-fine process significantly increases the parameters and computational cost through the concatenation of its sub-networks from the bottom to the top level. To mitigate this problem, U-Net was introduced to construct the network in [14,15,16], where U-Net serves as either the backbone or the sub-network. It has been presented that the U-Net-based networks can enhance the performance and reduce the memory and computational cost. However, the receptive field of CNN is inevitable limited due to the convolution operation, and the inductive bias is only focused on the locality as well. Therefore, the CNN-based networks cannot capture the long-range dependencies and global context of the images, which influences the deblurring performance.

It should be worth noting that the transformer [17] has been taken into account for computer vision tasks in recent research. The transformer is originally designed for sequence-to-sequence modeling in a natural language processing (NLP) community and has achieved tremendous success. Then, some works attempted to apply a transformer, rather than CNN, to process various vision tasks. For instance, vision transformer (ViT) [18] has acquired comparable performance on the task of large-scale image classification. The images are firstly sliced into non-overlapping patches. These patches are equipped with positional embedding to form a patch sequence, which is then processed using the global self-attention in the transformer. Similarly, a detection transformer (DETR) [19] leverages the transformer to explore the relationship between the objects and the global content of the image for object detection. The work of TransUNet [20] also employs the transformer as the encoder of U-Net for the task of image segmentation. 

In the aforementioned transformer-based methods, the mechanism of self-attention is employed to learn the correlation between different sliced patches so that the transformer can capture the long-range dependencies of images. Although transformer-based methods have been explored to some extent, there exists a restriction in the application of visual tasks, in that the transformer-based methods generally suffer from enormous computation costs when handling dense prediction at the pixel level. Specifically, the computational complexity of self-attention is quadratic to the image size, which restricts the application to high-resolution images. To reduce the tremendous computation cost, the Swin transformer [21] proposes the non-overlapping Window-based Multi-head Self-attention (W-MSA), where the self-attention is simply executed within non-overlapping local windows. In order to establish the relationship between the non-overlapping windows, the shifted window partition is exploited to bridge the windows of the preceding layer. In addition, the Swin transformer resorts to the layer of patch merging to effectively downsample the feature maps and construct the pyramid structure. By starting from small-sized patches and gradually merging neighboring patches in deeper layers, the Swin transformer sets up a hierarchical representation, which makes it feasible to combine it with U-Net for dense prediction. Therefore, it is of great significance to pay attention to the Swin transformer when constructing advanced networks for downstream tasks. In this work, we propose a novel deblurring network based on the W-MSA of the Swin transformer combined with a multi-input and multi-output U-shaped network (MIMO-UNet) [22]. We name this newly constructed network MIMO-Uformer, which takes advantage of the Swin transformer to improve the structure of MIMO-UNet for image deblurring in vehicle-surveillance scenarios.

The proposed MIMO-Uformer is constructed using the main architecture of the MIMO-UNet, of which the encoder and decoder blocks on different scales consist of the hierarchical Swin transformer instead of the CNN. The images are divided into non-overlapping patches, and each of the patches is rescaled based on the factors 1/2 and 1/4. By taking these patches in three different scales as inputs, the network can effectively extract the coarse and fine-grained feature representations of different semantic scales. Moreover, a Gated-Dconv Feed-Forward Network (GDFN) is adopted as the multi-layer perception (MLP) to further transform the features. However, there is a distinct feature of the motion blur in the vehicle-surveillance scenarios, where the motion blur is mainly caused by the only moving target instead of the shaking camera. The former brings a local blur, while the later causes a global blur. To improve the performance, two major efforts have been made as well. First, an IoP factor, which depends on the proportion of the blurry area overlapping with the input patches, is introduced to compensate for the training loss of local blur. Second, a supervised morphological loss is put forward as an auxiliary loss to enhance the restoration of the blur caused by the moving characters on the license plate. Benefiting from these improvements, the proposed MIMO-Uformer can effectively improve the quality of image deblurring for vehicle-surveillance scenarios. We evaluate the effectiveness of the proposed MIMO-Uformer based on the public GOPRO dataset [10]. Moreover, a motion blur dataset considering moving vehicles is established for further evaluation. To sum up, our main contributions are as follows:

(1) We propose a novel transformer-based network called MIMO-Uformer for image deblurring in vehicle-surveillance scenarios. The core design of MIMO-Uformer is to integrate the Swin transformer into MIMO-UNet as the basic block of the encoder and decoder sub-networks.
(2) We introduce an IoP factor to compensate for the loss values generated by the blurry patches. We also present a supervised morphological loss for the local deblurring of license plate regions. With these two improvements, the MIMO-Uformer can further enhance the performance on the image for vehicle-surveillance scenarios.
(3) We conduct extensive experiments on public, as well as self-established, datasets. It can be demonstrated that the proposed MIMO-Uformer is superiority to the other existing deblurring baseline, quantitatively and perceptually.

We note that a shorter conference version of this paper appeared in the work [23]. Our initial conference paper did not propose the new network of MIMO-Uformer. This manuscript introduces this novel architecture and provides an additional analysis using more data to demonstrate the efficiency and superiority. 

The rest of this article is organized as follows. Section 2 reviews the related works of coarse-to-fine CNN-based image deblurring methods and the application of vision transformers. The description of our proposed MIMO-Uformer, along with two improvements, is introduced in Section 3. Next, the extensive experiments and visualization analyses are executed in Section 4. Finally, Section 5 makes a conclusion of this whole work.

## 2. Materials and Methods

### 2.1. Image Deblurring Using Coarse-to-Fine Architectures

The CNN-based networks have made continuous progress in deblurring. One of the principal successful strategies is the coarse-to-fine framework, which uses multi-stage sub-networks to step-by-step recover the sharp image based on different resolutions. In this section, we review several coarse-to-fine architectures for image deblurring.

**Figure 1:**
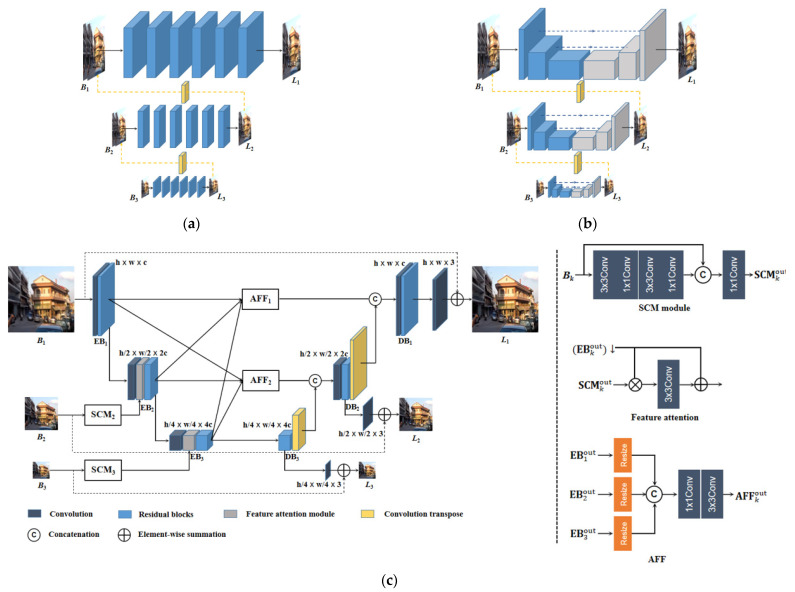
The structures of coarse-to-fine networks: (**a**) DeepDeblur. (**b**) PSS-NSC. (**c**) MIMO-UNet.

The pioneering work of coarse-to-fine networks is DeepDeblur [10], for which the architecture is shown in Figure 1a. The network is structured on three scale levels, and the ratio between the consecutive scale level is 1/2. In each level, several residual blocks are stacked to transform and maintain the resolution of the feature maps. The quadruple downsampled blurry patches *B*_3_ are first fed into the coarsest scale level to achieve the latent sharp image *L*_3_. Then, *L*_3_ is upsampled using an upconvolution layer and then concatenated with the blurry patches *B*_2_ to together feed into the second scale-level network. The structure of the second level is the same, with the coarsest scale level and the input features being processed the same as before. By this means, the sharp image *L*_1_ with the original resolution is restored at the finest scale level. Inspired by the success of DeepDeblur, a method containing parameter selective sharing and nest skip connection (PSS-NSC) is proposed in [16]. Similar with DeepDeblur, the main architecture of PSS-NSC is also composed of multi-scale levels as shown in Figure 1b. But there are two main distinct features in PSS-NSC in comparison with DeepDeblur. First, each sub-network is based on an encoder- and decoder-based U-Net where the feature maps can be directly transferred via the structure of symmetric skip connection. Second, because all the sub-networks are based on the same architecture of U-Net and on the same purpose to restore the image, most parameters of the sub-networks can be shared with each other. Consequently, the memory requirement of PSS-NSC is apparently reduced. In the architecture of multi-temporal recurrent neural networks (MT-RNNs) [24], the blurry image is formulated as an average of *M* clear images. The MT-RNN is stacked based on seven sub-networks and each is structured as a single U-Net. The process can be regarded as *M* decreasing with the sub-network, recovering until the clear image is generated from the last sub-network. Meanwhile, the feature maps from the decoder of the former U-Net are directly transferred to the encoder of the next U-Net. Owing to the repeated application of a single U-Net, MT-RNN further lowers the memory requirement. 

### 2.2. MIMO-UNet

It has been demonstrated that image blur can be better handled from multi-scale images [24]. Different from multiple stacked sub-networks, the multi-input multi-output U-shaped network (MIMO-UNet) [22] is mainly based on a single U-Net with multi-scale inputs and outputs. As shown in Figure 1c, the encoder and decoder are composed of encoder blocks (EBs) and decoder blocks (DBs) at three scale levels. In the finest level, the EB_1_ is used to extract the features from the blurry image B_1_. Then, the features from the finest level are downsized using a convolution layer with stride of 2 and combined with the second-level features extracted from the blurry images B_2_ using a feature attention module. The features at the second level are extracted using a shallow convolutional module (SCM), which consists of a couple of 3 × 3 and 1 × 1 convolutional layers. The output of SCM_2_ is element-wise multiplied with the downsized features from the finest level in the feature attention module. The fused features are considered to contain complementary information from different levels and then further refined by the following EB_2_. The process in the third level is similar to the second level. Moreover, MIMO-UNet utilizes asymmetric feature fusion (AFF) modules to fuse the outputs of EBs from different scales as shown in Figure 1c. The output of AFFs is transferred to the corresponding DB. Owing to AFF modules, DBs can exploit multi-scale features in a single U-Net, contributing to improving the deblurring performance. The intermediate supervision is executed to each DB, where the image restoration can be represented as

(1) $$L_{k}=\left\{\begin{array}{cc} o({\mathrm{DB}}_{k}{(\mathrm{AFF}}_{k}^{\mathrm{out}}{;\mathrm{DB}}_{k+1}^{\mathrm{out}}))+B_{k}, & n=1,2,\\ o({\mathrm{DB}}_{k}{(\mathrm{EB}}_{k}^{\mathrm{out}}))+B_{k} & n=3, \end{array}\right.$$

where ${AFF}_{n}^{out}$, ${EB}_{n}^{out}$, and ${DB}_{n}^{out}$ are the outputs of the n*^th^* level AFF, EB, and DB, respectively. The operation *o* is a single convolutional layer, which aims to generate an intermediate image *L*_k_ since the output of DB is a feature map, rather than an image. The content loss function *Loss*_cont_ is defined as follows:

(2) $${Loss}_{\mathrm{cont}}=\sum_{k=1}^{K}\frac{1}{t_{k}}{\left\|L_{k}-S_{k}\right\|}_{1},$$

where *K* represents the number of scale levels, and *S_k_* and *t_k_*, respectively, represent the sharp image and total number of image pixels under the *k^th^* scale level. The multi-scale frequency reconstruction (MSFR) loss is defined as follows:

(3) $$Loss_{\mathrm{MSFR}}=\sum_{k=1}^{K}\frac{1}{t_{k}}{\left\|\hat{F}(L_{k})-\hat{F}(S_{k})\right\|}_{1},$$

where $\hat{F}$ represents the FFT transformation of the image. The total loss function of the network is defined as

(4) $$Loss_{\mathrm{total}}=Loss_{\mathrm{cont}}+\lambda Loss_{\mathrm{MSFR}},$$

where *λ* is commonly set to 0.1. There are two versions with regard to the numbers of residual blocks in EB and DB, namely MIMO-UNet with 8 residual blocks and MIMO-UNet+ with 20 residual blocks.

### 2.3. Vision Transformer

**Figure 2:**
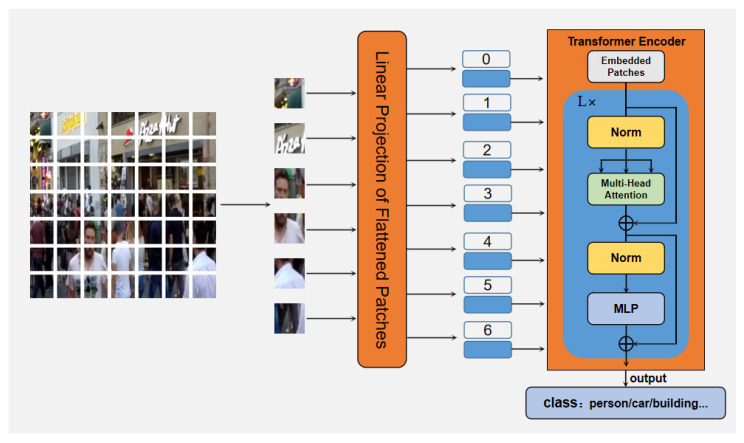
The illustration of Vision Transformer and Transformer Encoder.

The transformer is originally designed for natural language processing (NLP) where the transformer operates input sentences as a 1D sequence of token embeddings. The mechanism of self-attention enables the transformer to explore long-range dependencies in the data. Inspired by the success in NLP, numerous works have made efforts to use the transformer for various vision tasks, such as image classification [18], image recognition [25], segmentation [20,26], object detection [19], and image restoration [27]. Different from the CNNs, which suffer from an inherent inductive bias to the local receptive field, transformers employ a self-attention mechanism to make each pixel interact with all the other pixels, rendering a global receptive field and long-range dependencies [17]. In addition, the convolution filters in self-attention modules are estimated instantaneously with the input, surpassing conventional CNNs at the inference stage [28]. As shown in Figure 2, the Vision Transformer (ViT) directly slices the images into a sequence of non-overlapping medium-sized patches and receives patches as the token embeddings for image classification. To handle 2D images, an image with a size of *H* × *W* × *C* is first divided into *N* image patches with shape (*P*, *P*, *C*), where *N* = *H* × *W*/*P*^2^ is the number of patches and *P* is the patch size. Using a trainable linear projection, the patches can be flattened and transferred into a vector of patch embeddings with dimension *d*, which is constant through all of the layers. In order to retain the positional information, the patch embeddings add with the standard learnable 1D position embeddings E*_pos_*. The resulting sequence of embeddings serves as the input to the encoder. The standard vision transformer generally presents a plain architecture, which is constructed based on stacked identical encoders. There are two modules in each encoder. The former module is composed of a Layernorm (LN), followed by an multi-head self-attention (MSA), and the latter module is structured as an LN, followed by an multi-layer perception (MLP). In addition, a residual connection is employed for each module. The vision transformer can be formulated as

(5) $$\begin{array}{l} z_{0}{=[x}_{\mathrm{class}}{;x}_{p}^{1}E;x_{p}^{2}E;\ldots ;x_{p}^{N}{E]+E}_{pos},\;E\in {\mathbb{R}}^{(P^{2}\cdot C)\times d},\;E_{pos}\in {\mathbb{R}}^{(N+1)\times d}\\ z_{l}^{\prime }{=\mathrm{MSA}(\mathrm{LN}(z}_{l-1}{))+z}_{l-1},\;l=1\ldots L,\\ z_{l}=\mathrm{MLP}(\mathrm{LN}(z_{l}^{\prime }))+z_{l}^{\prime },\;l=1\ldots L,\\ y=\mathrm{LN}(z_{L}^{0}), \end{array}$$

where z’***_l_*** and z***_l_*** indicate the output of the MSA and MLP in the *l^th^* layer. In self-attention, the patch embeddings are transferred into queries Q, keys K, and values V with linear projections. The self-attention function is defined as

(6) $$\mathrm{Attention}(Q,K,V)=\mathrm{softmax}\left(QK^{T}/\sqrt{d}\right)V,$$

In the equation, the attention value is formulated as a weighted sum of values V, and the weights are computed as normalized element-wise products between Q and the transpose of K, and *d* is the factor used to avoid gradient vanishing. It is beneficial to respectively project Q, K, and V with different trainable linear projections and execute the attention function in parallel. Therefore, MSA split the output into *h* chunks along the channel dimension, and each chunk performs an independent group of linear projections. The formulation of MSA is illustrated as

(7) $$\mathrm{MSA}(X)=\mathrm{Concat}({\mathrm{head}}_{0},{\mathrm{head}}_{1},\ldots ,{\mathrm{head}}_{h})W^{o},\;W^{o}\in {\mathbb{R}}^{d\times d},\;{\mathrm{head}}_{i}=\mathrm{Attention}({\mathrm{XW}}_{i}^{q},{\mathrm{XW}}_{i}^{k}{,\mathrm{XW}}_{i}^{v}),\;{\mathrm{head}}_{i}\in {\mathbb{R}}^{H\times W\times d/h},\;{\mathrm{XW}}_{i}^{q},\;{\mathrm{XW}}_{i}^{k},\;{\mathrm{XW}}_{i}^{v}\in {\mathbb{R}}^{d\times d/h},$$

where head*_i_* is the output of the i*^th^* attention head. $W_{i}^{q}$, $W_{i}^{k}$, and $W_{i}^{v}$ correspond to the input linear projection, and W^o^ is used to group all heads. MSA has a complexity of O(*H*^2^*W*^2^), which is tremendous when handling dense predictions. Therefore, it is essential to design an effective transformer-based for dense prediction.

## 3. Proposed Method

### 3.1. Overview of Proposed Architecture

**Figure 3:**
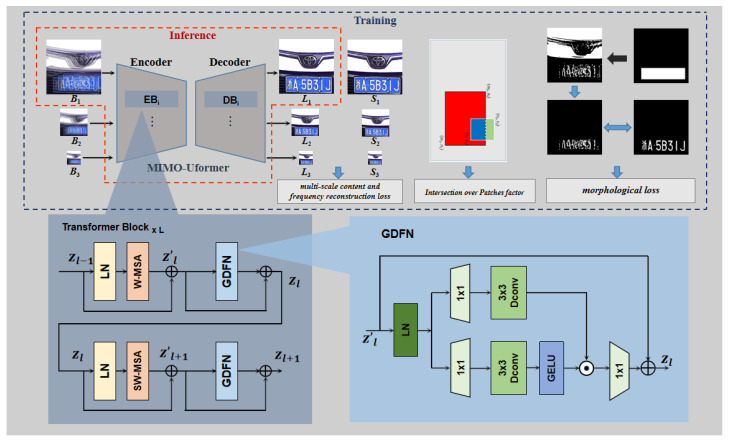
The architecture of the proposed deblurring method for vehicle-surveillance scenarios. The Chinese character on the plates means the province where the vehicle is registered.

The overview of the proposed deblurring method in vehicle-surveillance scenarios is shown in Figure 3. This network is mainly based on a novel designed transformer-based network, named MIMO-Uformer, which is developed from the architecture of MIMO-UNet. Concretely, the EBs and DBs are made up of stacks of transformer blocks, of which each mainly contains a W/SW-MSA and a GDFN module. During training, the image patches are first randomly cropped and rescaled based on factors of 1/2 and 1/4 to form a multi-scale sequence of blurry patches *B*_i_ (*i* = 1, 2, 3). Each *B*_i_ is fed into the corresponding EB_i_, and DB_i_ generates the latent sharp patch *L*_i_, which is combined with the label patch *S*_i_ to compute the content loss and the multi-scale frequency reconstruction loss as illustrated in Equations (2) and (3). Moreover, an Intersection over Patches, i.e., IoP factor, is proposed to compensate for the loss term generated by the moving vehicle. A supervised morphological loss is also introduced to enhance the restoration of the characters on license plates. Then, the network only receives *B*_1_ as input and exports *L*_1_ at the inference stage.

### 3.2. W/SW-MSA as the Basic Block

In the standard vision transformer, the value of each pixel is calculated based on the self-attention using all other pixels, which results in a quadratic computation complexity with regard to the image size. 

**Figure 4:**
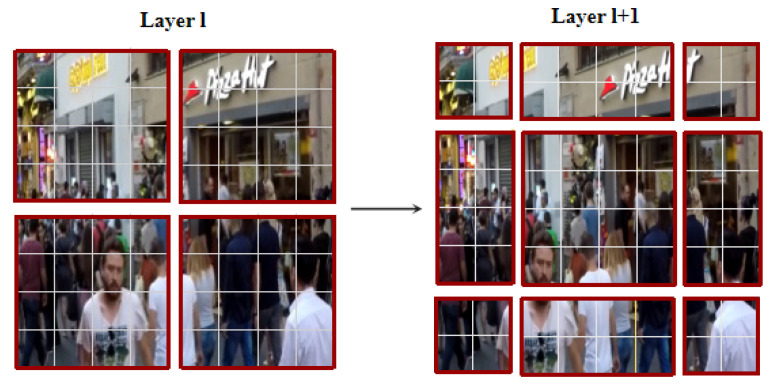
Regular local window partition and shifted window partition.

To overcome the problem, the Swin transformer introduces the mechanisms of window-based multi-head self-attention (W-MSA) and shift window-based multi-head self-attention (SW-MSA). Different from the global self-attention in the vanilla transformer, W-MSA first divides the feature maps into non-overlapping windows and then executes self-attention in each local window. As shown in Figure 4 (left), the feature map is partitioned into a sequence of non-overlapping square windows (with dark red edges). Each local window is flattened and transposed into a vector so that self-attention can be executed within the local window. Assuming the feature map with shape (*H*, *W*, *C*), the computational complexity is transferred from *O*(*H*^2^*W*^2^*C*) to *O*(*HW*/*M*^2^*M*^4^*C*) = *O*(*M*^2^*HWC*), where *M* means the size of the local window. Since the value of *M* is far smaller than the feature map size, the computational complexity is remarkably reduced by W-MSA. Although the operation of the window partition seems to restrict the self-attention to explore the long-range relationship, the pixels of feature maps at low resolution in U-Net have large receptive fields, which is sufficient to learn long-range dependencies. However, the execution of W-MSA is considered to hinder the interaction between the partitioned regions. To promote the contact across the windows, the Swin transformer introduces a shifted window partitioning strategy, which alternates two partitioning configurations in consecutive Swin Transformer blocks. As shown in Figure 4, in the *l*^th^ layer with a regular window partition, the feature map is sliced into several regular local windows with a size of *M* × *M*, within which the self-attention is calculated. In the *l* + 1th layer, the partition of the window is executed by shifting the windows by (*M*/2, *M*/2) pixels from the regularly partitioned windows in the preceding *l*^th^ layer. Then, the self-attention computation in the new windows crosses the boundaries of the previous windows in layer *l*, realizing connections among them. Therefore, the outputs of the process can be expressed as

(8) $$z_{l+1}^{\prime }{=\mathrm{SW}\text{-}\mathrm{MSA}(\mathrm{LN}(z}_{l}{))+z}_{l},\;z_{l+1}=\mathrm{MLP}(\mathrm{LN}(z_{l+1}^{\prime }))+z_{l+1}^{\prime },$$

where LN means the layer normalization in the network. We also apply the relative position bias to self-attention, which, computed in W-MSA and SW-MSA, can be expressed as

(9) $$\mathrm{Attention}(z_{l})=\mathrm{softmax}(QK^{T}/\sqrt{d}+B)V,$$

where B is a tensor with a dimension of (2*M* − 1) × (2*M* + 1) with learnable parameters. The SW-MSA is concatenated behind the W-MSA to form the two consecutive deblurring blocks as illustrated in Figure 3.

The traditional MLP is a feed-forward network (FN), which consists of two 1 × 1 convolution layers and a non-linearity GELU in the middle. The first convolution layer aims to expend the feature channels commonly with the factor of four, and the second is to restore the channel to an original dimension. To better transform the features, a Gated-Dconv Feed-Forward Network (GDFN) [29] serves as the MLP in the basic block of MIMO-Uformer. As shown in Figure 3, there are two distinct features, the namely gating mechanism and depth-wise convolution introduced to enhance the performance of the FN. The gating mechanism is structured as two parallel paths of linear transformation layers. In each path, a 3 × 3 depth-wise convolution is employed following the 1 × 1 convolution to capture the local context, which enhances the restoration of the local structures. Subsequently, one of the outputs is activated based on GELU non-linearity, and then element-wise multiplied with the other output. Given an input tensor X, GDFN is computed as

(10) $$X=W_{p}^{0}\mathrm{Gating}(X)+X,\;\mathrm{Gating}(X)=\mathrm{GELU}(W_{d}^{1}W_{p}^{1}(\mathrm{LN}(X)))⊙W_{d}^{2}W_{p}^{2}(\mathrm{LN}(X)),$$

where ⊙ denotes element-wise operation, and LN and GELU, respectively, mean the layer normalization and GELU non-linearity. It has been demonstrated that the GDFN controls the information flow through the hierarchical levels, which benefits detailed information complimentary to each other. Then, the process of the basic block of the MIMO-Uformer is formulated as

(11) $$\begin{array}{l} z_{l+1}^{\prime }{=\mathrm{SW}-\mathrm{MSA}(\mathrm{LN}(z}_{l}{))+z}_{l},\\ z_{l+1}=\mathrm{GDFN}(\mathrm{LN}(z_{l+1}^{\prime }))+z_{l+1}^{\prime },\\ z_{l}^{\prime }=\mathrm{MSA}(\mathrm{LN}(z_{l-1}))+z_{l-1},\\ z_{l}=\mathrm{GDFN}(\mathrm{LN}(z_{l}^{\prime }))+z_{l}^{\prime }, \end{array}$$

We use the framework of MIMO-UNet introduced in Section 2.2 as the main structure of our model. In each DB and EB, the CNN residual blocks are upgraded by the stacked transformer blocks, which are composed of W/SW-MSA and GDFN, as Equation (11).

### 3.3. Intersection over Patch Factor

**Figure 5:**
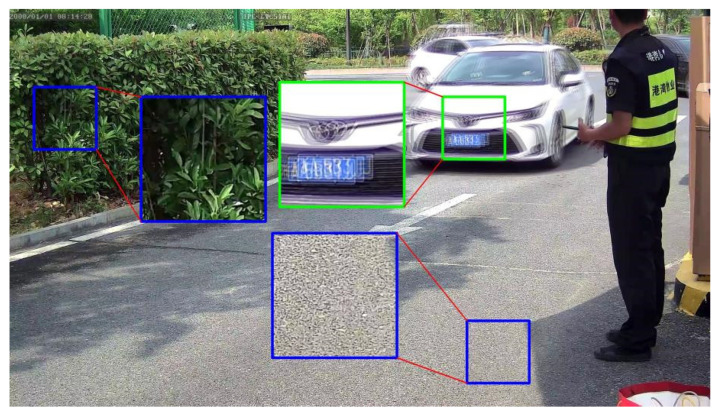
The illustration of the cropped patches.

For the most widely used datasets for image deblurring, such as GOPRO [10], RealBlur [30], and Kohler [31], the motion blur is mainly generated by the shaking of the camera, and little is produced by moving targets. It is obvious that the motion blur in these datasets is different from that in the vehicle-surveillance images where the motion blur is mainly caused by the moving vehicles. In training stage, a batch of randomly cropped rectangular patches, instead of the whole images, is fed into the network as the input. For the vehicle-surveillance image as shown in Figure 5, the randomly selected patches are more probably from the clear regions (blue blanks) than from the blurry region (green blank). These patches from the background hardly produce loss and thereby affect the back-propagation to update the network, leading to unpredictable convergence of the network. Therefore, more blurry patches are expected to be selected as the input.

An intuitive solution is to increase the number of the patches from the blurry regions as the input during every training step. However, it is difficult to determine the ratio between the blurry and clear patches inputted into the network. Besides restoring the blurry regions, the network is also expected to maintain the clear regions as unchanged. Excessive blurry patches result in the network over-fitting to the blur, and then, the restoration of the clear regions may achieve uncontrollable results. 

**Figure 6:**
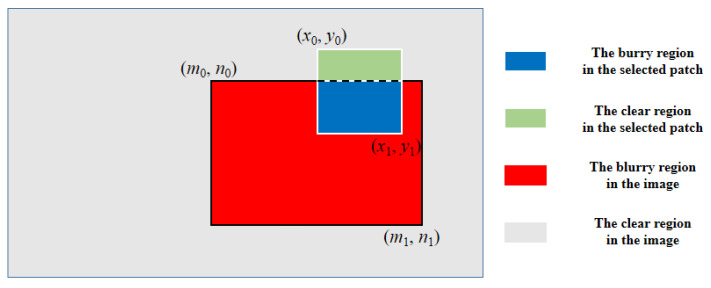
The illustration of the Intersection over Patch (IoP).

To solve the problem, a self-adaptive loss compensation factor based on the ratio of the areas of the blurry region in the input patch, called Intersection over Patch (IoP), is proposed. As illustrated in Figure 6, the square region (blue and green) with white boundaries stands for the selected patch, while the rectangular region (red and blue) with black boundaries represents the blurry region. The coordinates, represented as (*X*_min_, *Y*_min_, *X*_max_, *Y*_max_), of the patch and blurry region are respectively shown as (*x*_0_, *y*_0_, *x*_1_, *y*_1_) and (*m*_0_, *n*_0_, *m*_1_, *n*_1_). During training, the coordinates of the cropped patches are recorded and computed together with the provided coordinates of the blurry regions to calculate the IoP factors. The area of the intersection between the patch and blurry region is calculated as

(12) $$AR_{\mathrm{inter}}=W_{\mathrm{overlap}}\times H_{\mathrm{overlap}},\;W_{\mathrm{overlap}}=\max(0,\min(x_{1},m_{1})-\max(x_{0},m_{0})),\;H_{\mathrm{overlap}}=\max(0,\min(y_{1},n_{1})-\max(y_{0},n_{0})),$$

where the *AR*_inter_ corresponds to the area of the blue region in Figure 6. The *W*_overlap_ and *H*_overlap_ represent the overlapping length between the patch and blurry region along horizontal and vertical directions, respectively. Then, the *IoP* factor is expressed as follows:

(13) $$IoP=AR_{\mathrm{inter}}/AP,\;AP=(x_{1}-x_{0})\times (y_{1}-y_{0}),$$

where the *AP* means the area of the cropped patches. Finally, the loss function of the network is recalculated as

(14) $$Loss_{\mathrm{new}}=(1+IoP)\times (Loss_{\mathrm{cont}}+\lambda Loss_{\mathrm{MSFR}}),$$

In this expression, the patches near the moving vehicles usually possess more blurry regions and then yield larger values of the *IoP* and training loss. Meanwhile, the clear patches far from the vehicle produce smaller *IoP* factors but maintain the original training loss. The clear patches are still fed into the network to suppress the network over-fitting to the motion blur. Owing to this compensation, the network can restore the blur and maintain the clarity simultaneously.

### 3.4. Supervised Morphological Loss

**Figure 7:**
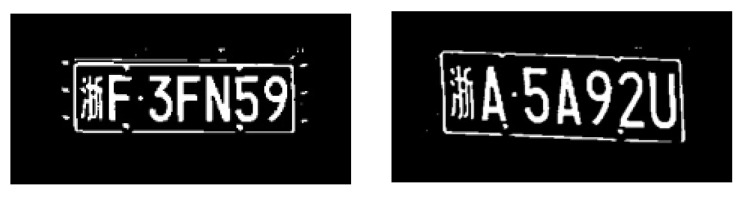
Examples of the binarization plates. The Chinese character on the plates means the province where the vehicle is registered.

The license plate, which contains a string of characters to uniquely identify the vehicle, is another important region to be restored in the vehicle-surveillance images. However, the region of a license plate is even smaller compared to the vehicle itself; therefore, the license plate is more difficult to be selected as the input patch. To enhance the performance of the restoration of this region, a supervised morphological loss is proposed using the morphological structure of the characters on the license plate. A binarization is performed on the plate to separate the characters from the background. The samples of the binarization plates with separate characters are shown in Figure 7.

When the decoder at the first level outputs the latent images, the binarization of the license plate is calculated simultaneously to compute the supervised morphological loss with the provided binarization labels, as shown in Figure 7. For convenience, a binary mask, where the pixels in the plate region are set to one while others are set to zero, is applied to separate the binarization result of the license plate. We execute the morphological loss at the first scale level. The loss function of the network is modified as follows:

(15) $$Loss_{\mathrm{total}}=(1+IoP)\times (Loss_{\mathrm{cont}}+\lambda_{1}\times Loss_{\mathrm{MSFR}})+\lambda_{2}\times Loss_{\mathrm{mor}},\;Loss_{\mathrm{mor}}={\left\|M(L_{1}^{\mathrm{plate}})-M(S_{1}^{\mathrm{plate}})\right\|}_{1},$$

where *Loss*_mor_ represents the supervised morphological loss, and *M* means the morphological binarization operation. **$L_{1}^{plate}$** and **$S_{1}^{plate}$** refer, respectively, to the plate regions of the latent and sharp images at the first level. We set *λ*_1_ = 0.1 and *λ*_2_ = 1 experimentally.

## 4. Results

This paper proposes an image deblurring network, called MIMO-Uformer, for the vehicle-monitoring images. To demonstrate the effectiveness, extensive experiments are carried out on public and self-collected datasets.

### 4.1. Dataset and Training Setting

The dataset GOPRO is most widely used for evaluating the deblurring performance. We first test on GOPRO to demonstrate the superiority of the architecture of MIMO-Uformer. There are commonly two ways to produce blur images. Previous works employ a blur kernel to convolve the sharp image, which leads to a global and unvarying blur. In GOPRO, the blurry images are generated by accumulating every sharp image during the exposure. In comparison, the latter is competent to make the moving targets blur and maintain the static background clear. However, the camera keeps shaking when collecting images, which results in global blur in the GOPRO dataset. Similarly, with GOPRO, the global motion blur in most of the other datasets, such as RealBlur and Kohler, is mainly caused by the shaking of the camera combined with slight motion of the targets. Reversely, the motion blur in vehicle-surveillance scenarios is primarily caused by the local moving cars. Therefore, we target collecting the data and establish a self-collected dataset of image blur from vehicle-monitoring scenes.

We fixed a camera on a Pan-Tilt to avoid the global blur caused by shaking and presented in the GOPRO dataset. The device is placed at the entrance to record the entering vehicles, which are commonly the family cars of the staff. We collect the video on a sunny morning to guarantee the clarity. The camera that we used has a resolution of 2880 × 1632 and a frame rate of 25 fps. We approximate the blur accumulation process using the *N* adjacent frames *S* as follows [10]:

(16) $$B=\frac{1}{N}\sum_{i=0}^{N-1}S[i],$$

**Figure 8:**
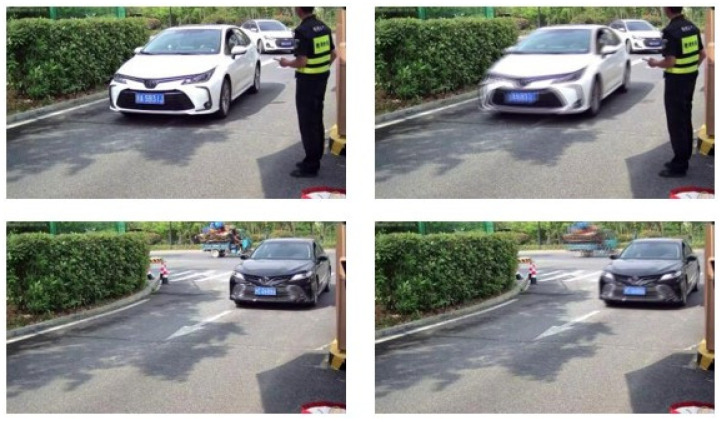
Examples of the sharp and blurry image pairs. The Chinese character on the plates means the province where the vehicle is registered.

We add the adjacent frames and take the average value to synthesize a blurry image and pick the middle frame as the sharp ground-truth. In this way, only the moving vehicles suffer from motion blur, while the background remains clear in the self-established dataset, as shown in Figure 8. We totally collected 25 videos, of which each records an independent vehicle passing the entrance. We selected 20–50 consecutive frames from each video and established a dataset containing 845 “sharp and blur” pairs of vehicles altogether, wherein 535 pairs made up from 16 videos are selected as the training set, and the left 310 pairs from 9 videos serve as the testing set. The ratio between the training and testing pairs are similar with the GOPRO dataset, of which the proportions of training and testing pairs are approximately 65% and 35%, respectively. In addition, the coordinates of the regions of the vehicle and license plate are also required in the proposed method. A well-trained object-detection model is implemented based on the self-collected images to output the coordinates of the vehicles and license plates. For every image, we execute binarization to the regions of the license plates to generate the supervised morphological labels.

We introduce two MIMO-Uformer variants in our experiments, MIMO-Uformer-S (Small) and MIMO-Uformer-B (Base), by setting different numbers of the Transformer blocks in each encoder and decoder stages. The details are listed as follows:

1. MIMO-Uformer-S: *C* = 32, depths of Encoder = {2, 4, 8};
2. MIMO-Uformer-B: *C* = 32, depths of Encoder = {8, 8, 8};

For every training iteration, we set the training batch as four patches with a size of 256 × 256, of which each is randomly cropped from a randomly selected image. Each patch is horizontally and vertically flipped with a probability of 50%. A total of 3000 epoches of training is directly carried out on the dataset of GOPRO. To deal with vehicle motion blur, we first pretrain the model on GOPRO for 500 epochs and then fine-tune it for another 2500 epochs based on the self-established dataset using the proposed network of MIMO-Uformer equipped with the IoP factor and supervised morphological loss. We use the cosine decay strategy to decrease the learning rate from the initial 1 × 10^−4^ to 1 × 10^−6^. The AdamW optimizer is adopted with the momentum terms of (0.9, 0.999) and the weight decay of 0.02. The experiments are conducted on a NVIDIA Tesla V100.

### 4.2. Comparative Experiments

We first evaluate the deblurring performance of MIMO-Uformer-S with the state-of-the-art CNN-based methods [10,16,22,24,32,33]. We also compare the MIMO-Uformer-S with its CNN counterparts, including the MIMO-UNet with 8 residual blocks and MIMO-UNet+ with 20 residual blocks in each EB and DB. The commonly-used Peak Signal-to-Noise Ratio (PSNR) and Structural Similarity Index Metrics (SSIMs) are adopted to assess the restoration performance. The definitions of these two metrics are shown as follows:

(17) $$\mathrm{MSE}=\frac{1}{MN}\sum_{i=0}^{M-1}\sum_{j=0}^{N-1}{\left(S[i,j]-L[i,j]\right)}^{2},\;\mathrm{PSNR}=10\times \log_{10}(\frac{{\mathrm{Peak}}^{2}}{\mathrm{MSE}})=20\times \log_{10}(\frac{\mathrm{Peak}}{\sqrt{\mathrm{MSE}}}),\;\mathrm{SSIM}(x,y)=\frac{\left(2\mu_{x}\mu_{y}+C_{1})(2\sigma_{xy}+C_{2}\right)}{\left(\mu_{x}^{2}+\mu_{y}^{2}+C_{1})(\sigma_{x}^{2}+\sigma_{y}^{2}+C_{2}\right)}.$$

where *S* and *L* are, respectively, the sharp and restored images. Peak is the maximum value of the image. *μ* and *σ* are the mean and standard deviation values. *C*_1_ and *C*_2_ are constant values to maintain the stability.

**Figure 9:**
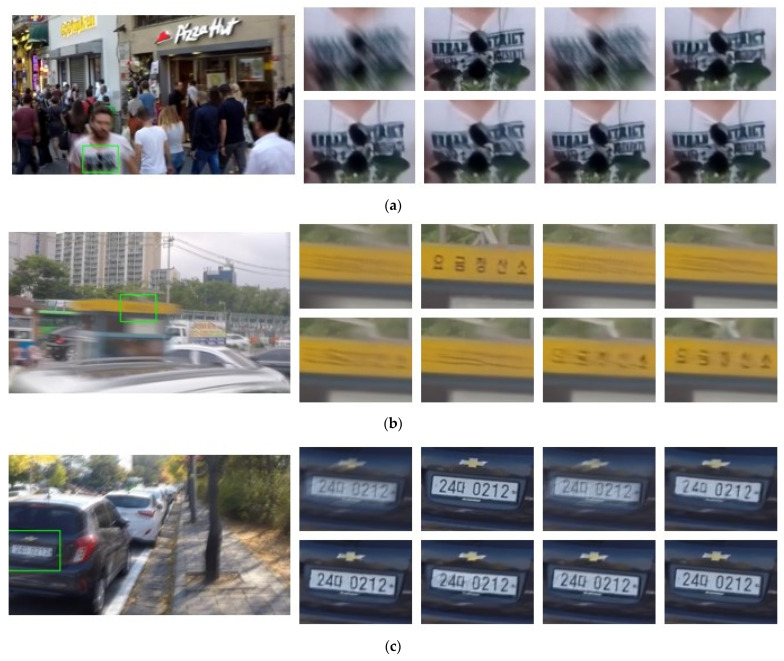
Examples of the deblurring results based on GOPRO. For clarity, the magnified parts of the resultant images are presented. The subfigures are the restorations of crowded people, traffic and roadside (**a**). From left-top to right-bottom: blur images, ground-truth images, and the resultant images obtained using DeblurGan, DeepDeblur, MT-RNN, MPRNet, MIMO-UNet+, and MIMO-Uformer, respectively. The Korean characters means the name of the shop in (**b**), and the region and usage of the vehicle in (**c**).

As shown in Table 1, the MIMO-Uformer-S achieves superior performance based on PSNR and SSIM compared with all the CNN-based methods. It can be evidently illustrated that the MIMO-Uformer-S largely boosts the performance based on the metrics compared to the traditional coarse-to-fine networks, including DeepDeblur, PSS-NSC, and MT-RNN. For example, MIMO-Uformer-S achieves better performance, respectively +3.75 dB and +0.050, based on PSNR and SSIM, but requires 5.9 M parameters fewer than the pioneering DeepDeblur. Despite using slightly more parameters, MIMO-Uformer-S generates obviously higher PSNR and SSIM than PSS-NSC (+2.06 dB and +0.024) and MT-RNN (+1.83 dB and +0.021), which both employ the standard U-Net as the architecture of the sub-network. Compared with MIMO-UNet+, the MIMO-Uformer-S exceeds 0.53 dB and 0.009 with respect to PSNR and SSIM respectively, and simultaneously significantly reduces the parameters from 16.1 M to 5.8 M, which demonstrates that the architecture of MIMO-Uformer-S surpasses its CNN counterparts. MPRNet is currently the best CNN-based deblurring network. Our proposed MIMO-Uformer-S produces outstanding PSNR (+0.32 dB) and SSIM (+0.007) but evidently lowers the numbers of parameters more than three times. In addition, our method requires the fewest MACs (based on image patches with the size of 256 × 256 pixels) compared with other methods, which illustrates that the structure of MIMO-Uformer-S is more efficient than the CNN-based networks. Apart from the quantitative metrics, MIMO-Uformer-S generates the optimum restoring results. We also reproduced the results based on GOPRO using the author-released network models. There are three groups of recovery results exhibited in Figure 9. The left is the completed blurry image with a green rectangle and the right are two columns of deblurring results of the rectangular regions using different methods. Although all the resultant images remain more or less blur compared with the GT, our method generates sharper results as displayed in the enlarged regions. Taking the first results as an example, the MIMO-Uformer-S and MIMO-UNet+ almost completely restore the characters on the shirt, while other methods still leave several residuals in the results. In the second example, although none of the methods effectively recovery the content, our proposed network achieves the sharpest results. It can be illustrated that the proposed architecture performs more outstanding perceptually.

We also made a comparison with other transformer-based deblurring architectures [15,29,34]. The MIMO-Uformer-B not only exceeds other transformer-based networks based on the metrics of PSNR and SSIM but also requires the fewest parameters and least computation costs. Due to the high-efficiency, the proposed MIMO-Uformer is more suited to the application based on mobile devices, where the resources of storage and computation are relatively restricted.

To verify the practicality, we conducted experiments on the self-established dataset based on vehicle-surveillance scenarios, where the images are acquired from security cameras. We equipped the MIMO-Uformer (Small) with an IoP factor and morphological loss, marked as MIMO-Uformer-Veh, to restore the moving vehicle. The comparative results with DeepDeblur and MIMO-UNet+ are exhibited in Table 2. The MIMO-Uformer-Veh outperforms both the DeepDeblur and MIMO-UNet+ based on PSNR (+1.92 dB and +0.74 dB) and SSIM (+0.0247 and +0.0008), which illustrates that the MIMO-Uformer-Veh is more efficient than other coarse-to-fine methods. MIMO-Uformer-Veh increases the PSNR by 0.16dB compared with original MIMO-Uformer. This improvement can demonstrate the effectiveness of the proposed IoP factor and the supervised morphological loss based on a moving vehicle dataset.

**Figure 10:**
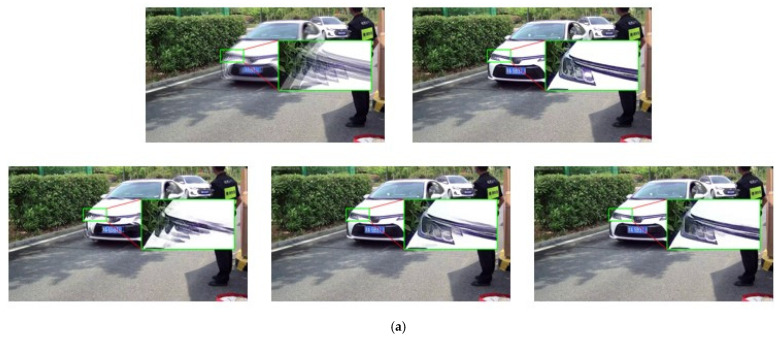

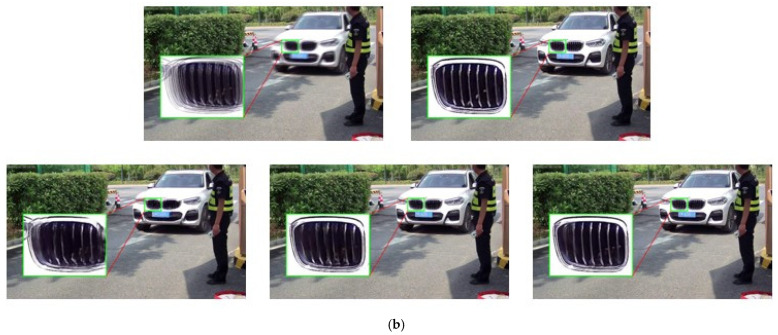
Examples of the deblurring results based on the self-established dataset. For clarity, the magnified parts of the resultant images are presented. The zoomed-in areas of subfigures are the restoration of lamp (**a**) and grille (**b**). From left-top to right-bottom: blur images, ground-truth images, and the resultant images obtained using DeepDeblur, MIMO-UNet+, and MIMO-Uformer-Veh, respectively. The Chinese character on the plates means the province where the vehicle is registered.

**Figure 11:**
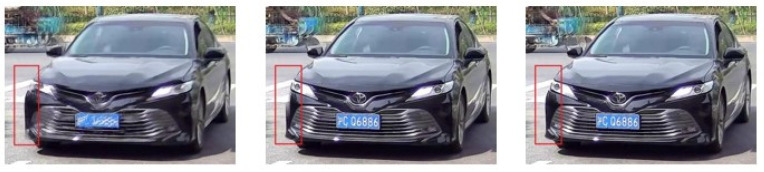
Examples of the deblurring results based on the self-established dataset. From left to right are the resultant images obtained using DeepDeblur, MIMO-UNet+, and MIMO-Uformer-Veh, respectively. The Chinese character on the plates means the province where the vehicle is registered.

Figure 10 shows two groups of delurring results for vehicle-surveillance scenarios, where sharp and blurry image pairs are presented in the first line, and the deblurring results of DeepDeblur, MIMO-UNet+, and the proposed MIMO-Uformer-Veh are displayed in the second line. The zoomed-in areas of the lamp and grille are also exhibited, respectively. As illustrated, the DeepDeblur method fails to handle the motion blur in this region. The MIMO-UNet+ also leaves some ghosting residue around the lamp region. By contrast, the proposed method shows the superiority by sweeping almost all motion blur in this region. Similarly, MIMO-Uformer-Veh presents superior performance based on the grille areas. Figure 11 displays another group of comparative results. There is still a mount of motion blur left in the results of DeepDeblur. Although the MIMO-UNet+ cleans up most of the blur, the vehicle is not restored completely and the region in the red blank is lost. The proposed MIMO-Uformer-Veh not only effectively removes the motion blur but also perfectly preserves the important detailed information of the car. Therefore, it can be demonstrated that the proposed method performs better than the existing methods from the subjective evaluation.

### 4.3. Ablation Experiments

The ablation experiments were respectively carried out to study some elements, including the IoP factor and the supervised morphological loss based on the motion deblurring for the vehicle-surveillance scenarios.

**Figure 12:**
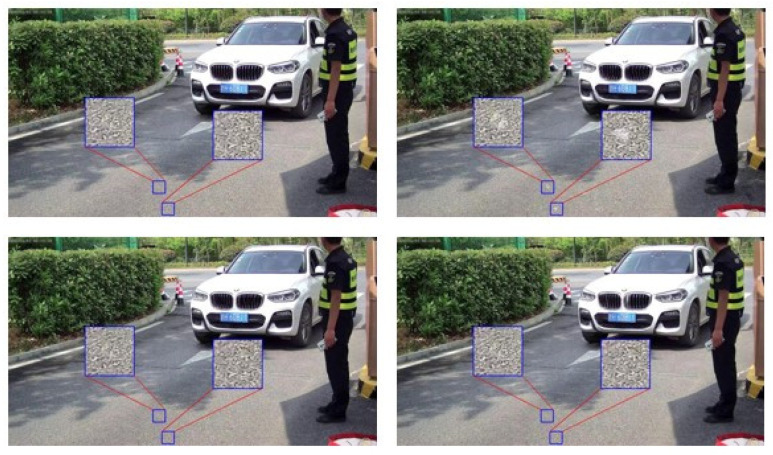
Examples of the deblurring results based on the self-established dataset. From left-top to right-bottom are the ground-truth image and resultant images obtained using MIMO-UNet+ (+R), MIMO-UNet+ (+I), and MIMO-Uformer (+I), respectively.

The methods of MIMO-UNet+ and MIMO-Uformer are respectively chosen as the benchmarks. The IoP-factor-based loss compensation and morphological loss are respectively added to MIMO-UNet+ to obtain MIMO-UNet+ (+I) and MIMO-UNet+ (+M) and MIMO-Uformer to obtain MIMO-Uformer(+M) and MIMO-Uformer (+I). From Table 3, it can be illustrated that either the IoP factor or the supervised morphological loss can enhance the performance. Concretely, the morphological loss contributes to improving the PSNR by 0.07 dB for MIMO-UNet+ and 0.09 dB for MIMO-Uformer, and the IoP factor increases the PSNR by 0.12 dB for MIMO-UNet+ and 0.11dB for MIMO-Uformer. It can be verified that both the IoP factor and morphological loss are beneficial for image deblurring for the vehicle surveillance-scenarios for different networks. We also conducted the experiment that all the training patches are cropped from the blur regions in the images. We used the MIMO-UNet+ and conduct the experiment that all the training patches are cropped from the blur regions in the images, which is marked as MIMO-UNet+ (+R). The performance based on PSNR and SSIM are, respectively, 27.65 dB and 0.9701 lower than the original MIMO-UNet+. Figure 12 represents a sharp frame and the deblurring results from its blurry counterpart using MIMO-UNet+ (+R), MIMO-UNet+ (+I), and MIMO-Uformer (+I). Some artifact appears in the zoomed-in clear regions and therefore weakens the performance of the MIMO-UNet+ (+R), which demonstrates that the introduced IoP not only improves the performance based on local deblurring but also maintains the sharpness based on clear regions.

**Figure 13:**
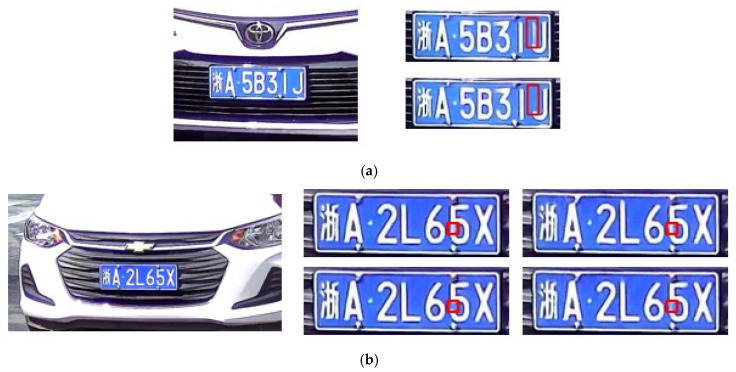
The subfigures display two deblurring results of two different license plates. In the first example, the ground-truth is illustrated in the left section, and the results are presented in the right section, of which the first and second row are the results of MIMO-UNet and MIMO-UNet (+M), respectively (**a**). In the second example, the ground-truth is illustrated in the left section, and the results are presented in the left section, of which the first row is the results of MIMO-UNet and MIMO-Uformer, and the second row is the results of MIMO-UNet (+M) and MIMO-Uformer (+M), from left to right (**b**). The Chinese character on the plates means the province where the vehicle is registered.

Figure 13 displays some deblurring results in the license plate regions. Overall, the methods without the supervised morphological loss achieve worse deblurring performance where “J” is mistaken for “U” and “5” is mistaken for “6”. By contrast, MIMO-UNet+ (+M) and MIMO-Uformer (+M) work better since the motion blur are thoroughly cleaned up and the characters are completely restored, which verifies the effectiveness of the morphological loss. A clear license plate is beneficial for recognizing the numbers for further processes [35].

Finally, we talk about the function of the pretrain for the transformer-based network. We conducted another training of MIMO-Uformer-Veh based on a self-established dataset, where the model is trained from scratch and obtain a PSNR of 25.85 dB, much lower than with pretraining (28.67 dB). By contrast, the MIMO-UNet+ without pretraining obtains a PSNR of 27.93 dB, higher than the 27.70 dB achieved using the model with pretraining. It has been demonstrated that the transformer-based models need large-scale datasets [36]. However, the self-established vehicle deblurring method is not large enough for the convergence of the model. Therefore, it is crucial for transformer-based network to pretrain the model based on a large deblurring dataset, such as the public GOPRO, and then fine-tune it based on the dataset of specific tasks.

## 5. Conclusions

In this paper, a transformer-based deblurring network, named MIMO-Uformer, is proposed for restoring the images under vehicle-surveillance scenarios. The main architecture of MIMO-Uformer is improved from a MIMO-UNet, where the blur is handled at multi-scale levels to enhance the deblurring performance. In the encoder and decoder blocks, the W-MSA and SW-MSA are exploited to explore the long-range dependencies while reducing the computational complexity of global self-attention. We also propose an IoP factor to compensate for the loss to improve the performance based on the local motion blur. Moreover, a supervised morphological loss is introduced to enhance the deblurring performance in the license plate region. Finally, we conduct extensive experiments based on the public dataset and a self-established dataset collected from the real vehicle-surveillance scenarios, based on which both comparative and ablation experiments are carried out. Based on a quantitative and perceptual evaluation, the structure of MIMO-Uformer is approved and superior to the existing CNN and transformer-based network, and the IoP factor and supervised morphological loss contribute to the deburring under vehicle-surveillance scenarios.

## Figures and Tables

**Table 1:** Comparative results based on GOPRO dataset with CNN and transformer-based methods.

	PSNR (dB)	SSIM	Params (M)	MACs (G)
DeblurGAN [32]	28.70	0.858	6.1	58
DeepDeblur [10]	29.23	0.916	11.7	336
PSS-NSC [16]	30.92	0.942	2.8	231
MT-RNN [24]	31.15	0.945	2.6	165
MPRNet [33]	32.66	0.959	20.1	777
MIMO-UNet [22]	31.73	0.951	6.8	67
MIMO-UNet+ [22]	32.45	0.957	16.1	154
Restormer [29]	32.92	0.961	26.1	155
Uformer [15]	33.06	0.967	51	86
Stripformer [34]	33.08	0.962	19.7	170
MIMO-Uformer-S	32.98	0.966	5.8	41
MIMO-Uformer-B	33.29	0.969	6.7	66

**Table 2:** Motion deblurring results based on self-established dataset under vehicle-surveillance scenarios.

	DeepDeblur	MIMO-UNet+	MIMO-Uformer	MIMO-Uformer-Veh
PSNR (dB)	26.75	27.93	28.51	28.67
SSIM	0.9479	0.9718	0.9717	0.9726

**Table 3:** Motion deblurring ablation results based on self-established dataset under vehicle -surveillance scenarios.

	MIMO-UNet+	MIMO-UNet+ (+M)	MIMO-UNet+ (+I)	MIMO-UNet+ (+I+M)
PSNR (dB)	27.93	28.00	28.05	28.09
SSIM	0.9718	0.9719	0.9720	0.9720
	MIMO-Uformer	MIMO-Uformer (+M)	MIMO-Uformer (+I)	MIMO-Uformer-Veh
PSNR (dB)	28.51	28.60	28.62	28.67
SSIM	0.9717	0.9725	0.9722	0.9726

## Data Availability

The original contributions presented in this study are included in the article. Further inquiries can be directed to the corresponding author.
